# Metagenomic next-generation sequencing may assist diagnosis of cat-scratch disease

**DOI:** 10.3389/fcimb.2022.946849

**Published:** 2022-09-16

**Authors:** Mingxia Li, Kunli Yan, Peisheng Jia, Erhu Wei, Huaili Wang

**Affiliations:** Department of Pediatrics. The First Affiliated Hospital of Zhengzhou University, Zhengzhou, China

**Keywords:** Bartonella henselae, Cat-scratch disease, mNGS, diagnosis, infection

## Abstract

*Bartonella henselae*, the pathogen that causes cat-scratch disease (CSD), is relatively rare in the clinic. CSD usually causes mild clinical manifestations, which self-heal in a matter of weeks. However, in immunocompromised patients, CSD may cause systemic disorders that can lead to critical illness. Due to the diversity of symptom signs and the lack of a golden standard for diagnosis, identifying atypical CSD in a timely manner presents a challenge. Metagenomic next-generation sequencing (mNGS), is a promising technology that has been widely used in the detection of pathogens in clinical infectious diseases in recent years. mNGS can detect multiple pathogens quickly and accurately from any given source. Here, we present a case of atypical CSD, which was diagnosed using mNGS. The patient manifested a fever of unknown infectious origin, and routine antibiotic treatment was ineffective. mNGS was employed to test the patient’s peripheral blood, which led to the detection of *B. henselae*. This was rarely seen in previous CSD reports. We surmised that the patient presented with atypical CSD and thus a targeted therapy was recommended. Crucially, the patient recovered rapidly. Based on this case study findings, we recommend that CSD should be included in the differential diagnosis for fever of unknown origin and that mNGS may be helpful in the diagnosis of CSD.

## Introduction

The three most common Bartonella species (spp.) infecting humans include: *Bartonella henselae*, which causes cat-scratch disease (CSD); *Bartonella bacilliformis*, which causes Carrion’s disease; and *Bartonella Quintana*, which causes trench fever. CSD is the most common human infection caused by Bartonella spp. *B. henselae* are small, fastidious, hemotropic, gram-negative intracellular bacteria (Pennisi et al., 2013; Nelson et al., 2016). Cat fleas are thought to be the main vector of *B. henselae* (Im et al., 2013). CSD is commonly diagnosed in children and teenagers (Karski et al., 2018). In the United States, the average annual incidence of CSD is about 4.5/100,000 for outpatient diagnoses and 0.19/100,000 for inpatient admissions (Nelson et al., 2016). *B. henselae* infection in humans has been reported worldwide but been rarely in China. In general, immunocompetent patients present with typical CSD, while immunocompromised patients often have an atypical form. The typical clinical manifestation of CSD occurs a few days after coming into contact with a cat. Initially, the patient develops erythematous papules at the infection site, after which the wound usually heals itself without leaving any scars. About two weeks later, regional lymphadenopathy occurs near the site of infection, which is the hallmark of the disease and is accompanied by nonspecific symptoms such as low-grade fever, headaches, loss of appetite, and fatigue. All symptoms normally disappear within a few weeks. However, 5−20% of patients develop atypical forms of the disease manifested by Parinaud’s oculoglandular syndrome, hepatosplenic abscesses, bacillary angiomatosis, bacteremia, fever of unknown origin, endocarditis, encephalitis, uveitis, conjunctivitis, osteitis, arthropathy, or myalgia, among others (Mazur-Melewska et al., 2015; Nawrocki et al., 2020).. Atypical CSD can cause the rapid deterioration of patients in a matter of days (Lemos et al., 2021). Therefore, such cases of CSD rely on a rapid and reliable diagnosis as well as an effective treatment regimen. However, due to the great variability in the signs and symptoms, as well as the lack of gold standard diagnostic criteria, the diagnosis of atypical CSD can be difficult and is often significantly delayed (Sodini et al., 2021).

## Case presentation

A 13-year-old boy was admitted to our hospital due to an intermittent fever for 12 days. Prior to this, the patient was perfectly healthy. It was confirmed by the patient and his relatives, that he had recently been in contact with a kitten but not with pigeons, sheep, or other animals. Prior to fever onset, the patient experienced some shivers and then went on to develop headaches during the fever episodes, a poor appetite, and weight loss. He had been given various antimicrobial drugs before being admitted to our hospital, but none of them proved effective. The boy’s temperature gradually rose to 40.2 °C, and the feverish episodes became more frequent; thus, the patient’s relatives asked for him to be admitted to our hospital.

According to the admission records, the patient initially had a temperature of 38.2 °C, with the fever occurring once a day. The patient experienced no other discomfort such as a cough, vomiting, diarrhea, rash, arthralgia, headache, or convulsion. Laboratory examination (performed in a local hospital) showed that the patient’s complete blood count and the immune function were normal. However, they did detect an increase in the serum C-reactive protein (CRP) levels to 54.08mg/L (normal 0−5 mg/L) and in the erythrocyte sedimentation rate (ESR) to 23 mm/h (normal 0−20 mm/h). Chest computed tomography (a CT scan) showed the presence of small nodules on the upper lobe of the right lung and on the lower lobes of both lungs, with a maximum diameter of about 4 mm. An ultrasound detected splenomegaly; the spleen was about 126 mm long and 40 mm thick. Etiological examination revealed no abnormalities, including mycoplasma, respiratory syncytial virus, Epstein-Barr virus, or Coxsackie virus. A lumbar puncture was performed, but no abnormality was detected in the patient’s cerebrospinal fluid.

At admission, the physical examination identified no positive signs. Although the ultrasound performed at the local hospital showed an enlarged spleen, it was not palpable at the left costal margin. The laboratory investigations on the first day of admission showed a significant increase in serum CRP (94.73 mg/L), and an ESR of 78 mm/h. The procalcitonin concentration was 0.285 ng/mL (normal 0−0.02 ng/mL). Other laboratory tests, including complete blood count, blood biochemical examination, coagulation test, and thyroid function, were all within the normal range. All other laboratory test results also came back negative; these included HIV infection, tuberculosis, other viral infections (e.g., cytomegalovirus, herpes simplex virus, hepatitis virus, influenza virus, among others), galactomannan test, connective tissue disease antibody test, tumor markers, two sets of blood cultures for the detection of aerobic and anaerobic bacteria, and bone marrow cytology and culture tests. Furthermore, echocardiography, a chest CT scan, and cranial magnetic resonance imaging (MRI) failed to detect noticeable abnormalities.

During the initial phase of treatment, the patient received empiric antimicrobial drugs such as latamoxef sodium, rifamycin, and penciclovir for four days. However, the fever persisted and the CRP levels and ESR rose to 107.58 mg/L and 98 mm/h, respectively. On the fifth day after admission, metagenomic next-generation sequencing (mNGS) was performed on the patient’s peripheral blood, using an Illumina Next 550 sequencer with a single-end 75 base pair sequencing strategy. A total of 19,446,948 reads were generated within 24 h. After quality control, 19,186,121 were aligned with the human reference genome (HG38) and 260,404 reads were used for downstream analysis. Finally, four *B. henselae*-specific sequences were detected. Since neither our hospital nor the Henan Center for Disease Control could perform serological antibody tests or polymerase chain reaction (PCR) for *B. henselae*, we carried out another blood culture but this time extended the culture period to six weeks. The patient’s treatment plan was adjusted, and the following antibiotics were administered: azithromycin (500 mg on the first day and 250 mg on the second to fifth days) together with rifampin (20 mg/kg/d, given in two doses), followed by doxycycline (200 mg/d, given in two doses) with rifampin. The fever gradually subsided and the shivering and headaches eventually disappeared. The patient’s CRP levels also decreased and his temperature returned to normal on the 17^th^ day after admission. The patient was feeling well enough to be discharged on the 19^th^ day. He continued to take doxycycline and rifampin orally for the entirety of the one-month-long course. When the patient returned to our hospital for a follow-up reexamination, he was in good health. No abnormalities were detected in his blood after the following tests were performed: complete blood count, CRP, ESR, procalcitonin concentration, blood biochemical examination, and mNGS. We were therefore able to discontinue the patient’s treatment. The patient’s follow-up prolonged blood culture remained negative for *B. henselae*.

## The criteria for CSD diagnosis

Although CSD was originally described in France in 1950, its cause was unknown until 1983, when the pathogenic bacterium was detected in the lymph nodes of a patient with CSD using a Warthin-Starry silver stain (Margileth, 2000). However, the ambiguity in the symptoms and signs of CSD renders the diagnosis of atypical forms of the disease challenging. When CSD is highly suspected based on the patient’s clinical manifestations and epidemiological background, sensitive and reliable laboratory tests must be used to accurately diagnose the disease and rapidly initiate appropriate treatment. A CSD diagnosis is generally derived from a history of animal exposure, and the presence of skin lesions, fever, lymphadenopathy, as well as the results of immunoserologic laboratory tests, *in vitro* culture, or DNA amplification methods (Chen et al., 2007). Margileth et al. (Margileth, 2000) proposed that CSD be diagnosed according to the following criteria: 1. Contact with cats or fleas, with or without the presence of a scratch mark or a regional inoculation lesion. 2. Laboratory/radiology testing: negative purified protein derivative or serology for other infectious causes of adenopathy; sterile pus aspirated from the node; positive PCR result. CT scan: liver/spleen abscesses. 3. Positive enzyme immunoassay (EIA) or indirect fluorescent serological antibody test >1:64 for *B. henselae*; a fourfold rise in titer between the acute and convalescent specimens is definitive. 4. Biopsy of lymph node, skin, liver, bone, or eye granuloma shows granulomatous inflammation compatible with CSD; Warthin-Starry silver stain indicates a positive result. However, given the patient’s medical history, the reliability of laboratory tests, and the difficulty of obtaining histopathology data, it is almost impossible to meet all these criteria in a patient simultaneously.

## Implementation of the criteria

Although contact with a cat is ubiquitous, only 60% of patients with CSD may have skin lesions caused by scratching or biting (Ulug, 2015). Serological tests, such as the EIA or indirect fluorescent antibody (IFA) assays, are the most popular laboratory methods for the diagnosis of CSD. These tests have high sensitivity (88%) and specificity (97%), but they may lead to incorrect results in a considerable number of cases (Vermeulen et al., 2007). Although immunoglobulin (Ig)M antibodies suggest an acute infection, the short duration in the blood may lead to false-negative results (Podsiadly et al., 2009; Johnson et al., 2020). IgM antibodies against *B. henselae* are seldom detected even in the early stages of CSD, and these negative results do not exclude the presence of acute disease (Ridder et al., 2002). Evidence of cross-reactivity has been observed between *B. henselae* infection and infection with other bacterial species such as *Coxiella burnetii*, *Chlamydophila* spp., and non-*henselae Bartonella* spp. These pathogens are common in healthy individuals, which may give rise to false-positive results. Besides, the IgG antibody response against *B. henselae* may remain positive one year after the initial infection. If the IgG titer is between 1:64−1:256, it must be rechecked at 10−14 days to confirm (Klotz et al., 2011). A PCR test performed on blood or tissue samples can detect different Bartonella spp. with a high degree of specificity. Meanwhile, PCR sensitivity is estimated to be much lower (40−75%) (Dona et al., 2018; Salmon-Rousseau et al., 2021), and the time point at which the pathogen can be detected is unknown (Yanagihara et al., 2018). It has been reported that the PCR technique fails to detect the pathogen in atypical CSD (Sarno et al., 2021). In addition, the PCR test for *B. henselae* is only available in specialist research laboratories and is costly. Pathological examinations are often delayed or rejected by the child patient’s parents (Sodini et al., 2021), and this method can’t be realized for patients without clear local lesions. Warthin-Starry silver staining is also unreliable for providing an accurate diagnosis of atypical CSD as it detects other bacteria such as *Helicobacter pylori* and *Legionella pneumophila*. Since *B. henselae* are fastidious, slow-growing, and difficult to isolate in the culture medium under most laboratory settings (Lamps and Scott, 2004), culture methods are generally not used for the diagnosis of CSD (Ulug, 2015).

The patient in this case study presented with a fever of unknown infectious origin, accompanied by nonspecific symptoms such as a headache, loss of appetite, and weight loss. Although the patient did have contact with a kitten, no other symptoms or signs such as lymphadenopathy, papules, impaired vision, liver and spleen abscess, encephalitis, endocarditis, etc. were detected. Because the patient had no local lesion, a pathological examination could not be performed. Relevant serological and PCR tests were also not available at our hospital or laboratory sites. Moreover, the blood culture results were negative. Considering the bacteremia caused by *B. henselae*, we prolonged the blood culture time, but still obtained nothing. Therefore, if we had to rely on these conventional detection methods, the etiology of the present case would have remained unknown.

## The use of mNGS for the diagnosis of CSD and other rare, atypical, and complex infections

As a fast and unbiased method, mNGS enables researcher to address open-ended questions about pathogens without potential assumptions. mNGS can simultaneously detect multiple pathogens in a given tissue, making this technique especially suitable for detecting pathogens with rare, atypical, and complex infections (Miller et al., 2018).

In the study by Wang et al. the conventional bacterial culture result of a patient with subcutaneous abscess was negative, while mNGS was able to detect *B. henselae* (Wang et al., 2020). In this particular study, the patient had been scratched by a cat one week before admission. On obtaining the mNGS result, the patient received a two-week course of doxycycline, and he was cured following abscess drainage and antibiotic treatment. Yang et al. found that hemophagocytic lymphohistiocytosis was associated with *B. henselae* infection in a patient with multiple susceptibility genes (Yang et al., 2020). The clinical manifestation and laboratory examination of the patient did not show any evidence of initial infection. Histopathological examination of lymph nodes suggested that it could be CSD, and subsequent mNGS analysis of the lymph node detected the presence of *B. henselae*. Patel et al. detected *B. henselae* in a patient with blood-culture-negative endocarditis, presenting with crescentic glomerulonephritis (Patel et al., 2022). The combination of microbial cell-free DNA NGS and serological methods confirmed *B. henselae* infection. Degner et al. detected *B. henselae* in 22 cases using plasma microbial cell-free DNA NGS, of which 12 cases were diagnosed as having CSD/fever of unknown origin (Nicholas Degner, 2021).

Xing et al. diagnosed seven patients with cerebral aspergillosis (a rare but often fatal, difficult to diagnose opportunistic infection) using mNGS of cerebrospinal fluid (Xing et al., 2021). This finding indicated that mNGS facilitates the diagnosis of cerebral aspergillosis and may reduce the need for invasive cerebral biopsy in patients with suspected cerebral aspergillosis. Chen et al. diagnosed visceral leishmaniasis in three patients using bone marrow mNGS (Chen et al., 2020). These patients were initially misdiagnosed and unsuccessfully treated for 2−6 months, indicating that the sensitivity of bone marrow mNGS was higher than that of smear microscopy in patients with suspected leishmaniasis. Xie et al. diagnosed a rare case of intracranial hemorrhage caused by Seoul virus infection using mNGS, when other conventional microbiological tests returned negative results (Xie et al., 2021). This study has exposed the various extrarenal manifestations of Seoul virus infections, which should not be overlooked. Chen et al. diagnosed nine cases of severe psittacosis pneumonia using mNGS (Chen et al., 2020). The clinical condition of these patients gradually deteriorated during the empirical treatment. The initiation of post-mNGS minocycline therapy led to a rapid recovery. The authors claimed that, mNGS shortened the time to diagnosis and enable the earlier initiation of targeted antibiotic therapy. Xie et al. used mNGS to diagnose a case of co-infection pneumonia with *Mycobacterium abscessus* and *Pneumocystis jiroveci* in a patient without HIV infection (Xie et al., 2021). Clinical progress was satisfactory following initiation of combined antifungal and anti-*M. abscessus* therapy. The research of Wang et al. showed that mNGS was an effective method for the detection of pulmonary fungal infection, but was not suitable for diagnosing cryptococcal pneumonia (Wang et al., 2020). Tang et al. reported the use of mNGS for the diagnosis of infection in 16 patients with primary immunodeficiency (Tang et al., 2021). The patients’ cerebrospinal fluid, bronchoalveolar lavage fluid and blood samples were subjected to mNGS and the results demonstrated that mNGS had a marked advantage over conventional methods in identifying infectious agents, especially suitable for detecting pathogens of opportunistic and mixed infections. Qian et al. also recommended the timely use of mNGS in cases when infection by mixed or rare pathogens was suspected, especially in immunocompromised individuals or individuals with severe conditions that required urgent treatment (Qian et al., 2020). However, almost all the aforementioned studies are retrospective case reports and small case series. The clinical utility of mNGS has not yet been established in large-scale prospective clinical trials. Furthermore, universal reference standards and robust test validation approaches for clinical metagenomic assays are lacking (Chiu and Miller, 2019).

Our case study documents the first reported case of *B. henselae* being detected in the peripheral blood by mNGS in China. Although mNGS is more rapid than bacterial culture methods and is less affected by antibiotic treatment or the bacterial growth conditions, there are few reports of its application to diagnose bacterial infection in the bloodstream. The reasons may include: 1. Unlike the large-scale replication of viruses, the load of bacteria in the blood is low. As the DNA of patients and pathogens needs to be detected simultaneously, its relative quantity may directly affect the method’s sensitivity. 2. As for the blood culture method, the risk of sample contamination during mNGS should be considered. For instance, to determine whether the flora residing on normal skin or in an oral cavity is a contaminant or a true pathogen may require at least two sets of blood samples from separate venipuncture sites. 3. The optimal sample volume is still unknown. 4. mNGS results are among the most challenging types of clinical laboratory data to interpret (Greninger and Naccache, 2019). In addition, antimicrobial susceptibility cannot be fully tested, which is very important for doctors to make antimicrobial treatment plans for patients. Collectively, these limitations may explain why mNGS is not routinely used for the detection of bacterial infection in the bloodstream.

## Interpreting mNGS results

There is no standard method for interpreting mNGS results. It is necessary to consider factors such as the quality of the test process and sample, the rarity of the pathogen and its relative and absolute abundance (Gu et al., 2019). Due to the complexity of mNGS methodology, contamination can be easily introduced at each step. It is often difficult to determine whether the detected pathogens are colonizing, background, or pathogenic forms of bacteria. Therefore, systematic analyses, interpretation and validation of laboratory and auxiliary examination results are necessary (Nan et al., 2022). In particular, the discovery of any atypical or novel infectious agent in clinical samples should be followed up with confirmatory investigations (e.g., tissue biopsies or seroconversion) to ascertain its true pathogenic nature (Chiu and Miller, 2019). For instance, Xing et al. considered that although the abundance of aspergillus might be low in the samples assayed by mNGS, it should not be regarded as background contamination because of the high mortality rate associated with aspergillus infection (Xing et al., 2021). Pathogen identification should be considered in the clinical context and in conjunction with the patient’s symptoms and signs, radiographic evidence, and the results of clinical laboratory tests (e.g., smear, culture, and galactomannan tests). In this regard, although pulmonary infection caused by *Neisseria meningitidis* may be uncommon, clinicians should still consider it as a potential causal pathogen of lung infection in immunodeficient patients. Therefore, clinicians need to correlate clinically to make accurate judgments when interpreting reports (Zheng et al., 2021). Pathogens typically considered as contaminants (e.g., *Bacillus cereus*) may still cause severe disease and should not be ignored when interpreting the mNGS results (Tusgul et al., 2017). Some institutions have established precision medicine teams composed of research experts in fields such as medical microbiology, infectious diseases, computational biology, and other clinician groups to discuss mNGS results and provide reasonable explanations (Mongkolrattanothai et al., 2017). The 2020 Expert Consensus on the Clinical Application of the Second-generation Sequencing Technology of Chinese Metagenomics to Detect Infectious pathogens puts forward that, when the results of mNGS are consistent with the patient’s clinical manifestations and relevant laboratory results, the mNGS results can guide the antimicrobial treatment. If the mNGS result is positive and meets the clinical criteria, but there is no other supporting laboratory evidence, PCR should be carried out with appropriate primers. In addition, it is suggested that the available conventional laboratory tests are further improved for validation purposes. In circumstances when the mNGS result is positive, but the clinical manifestations or laboratory tests does not support this result, it is recommended that conventional laboratory tests are relied upon. For patients whose mNGS results are negative but are highly suggestive of possible infection according to other examination results, such as culture methods, it is recommended to take another sample and repeat the mNGS test (C Q et al., 2020).

For the patient in this case study, mNGS was performed on the fifth day after hospital admission. The result only showed the presence of *B. henselae*, which led us to suspect CSD. We were aware that the patient had contact with a kitten and that atypical CSD could manifest as bacteremia with a fever of unknown origin. There was no laboratory evidence to support the infection by other pathogens, and the patient failed to respond to empirical treatment. The absence of specialist test centers nearby means that serological and PCR tests were not carried out. Furthermore, identification of *B. henselae* by prolonged blood culture time proved fruitless. After being given the targeted antimicrobial treatment, the patient’s clinical symptoms disappeared and the laboratory indicators improved rapidly, thus validating the mNGS results.

## Antibiotic treatment of CSD

Due to the uncertain effect of antibiotics in atypical CSD and the lack of official recommendations, the clinical application of antibiotics is usually based on personal experience and expert opinions (Salvatore et al., 2015). Although antibiotics are not recommended for the treatment of mild to moderate cases of typical CSD, they are recommended for treating atypical CSD, especially in immunocompromised patients. Antibiotics that have been approved include azithromycin, rifampicin, doxycycline, ciprofloxacin, sulfamethoxazole, and gentamicin (Margileth, 1992; Rolain et al., 2004). The combined use of azithromycin and rifampin is a common treatment choice, although the dose and course of treatment vary widely (Johnson et al., 2020). The administration of 5−12 mg/kg/d azithromycin once daily or rifampin 20 mg/kg/d in two doses for 2−3 weeks have been shown to be effective (Margileth, 1992). Dornbos and colleagues have reported switching to intravenous doxycycline and rifampin when azithromycin treatment failed in a rare case of multifocal thoracic osteomyelitis with an epidural abscess (Dornbos et al., 2016). This decision was based on the limited published data relating to *B. henselae*-associated vertebral osteomyelitis in adults, as well as studies reporting the treatment of other serious *B. henselae* infections (Rolain et al., 2004). Immunocompetent patients over the age of eight can be treated with doxycycline (200 mg/d given in two doses for 2−4 weeks), which can be delivered intravenously or in combination with rifampicin. For immunocompromised patients, the treatment regimen needs to be extended to four months (Cunningham and Koehler, 2000). Of note, a clinical study showed that patients with eye lesions treated with antibiotics combined with oral glucocorticoids had a better final outcome (i.e., restoration of vision) than those treated with antibiotics alone (Habot-Wilner et al., 2018).

## Conclusion

The diagnosis of atypical CSD is clinically challenging. This case study highlights the need to carefully evaluate and screen the complex and diverse clinical manifestations of a given disease to avoid delaying diagnosis and risking patient deterioration. Even in patients lacking the typical characteristics of CSD, this disease should be included in the list of differential diagnosis in patients with fever of unknown origin. When a lymph node biopsy is not feasible or serological results are uncertain, mNGS should be considered as a method for diagnosing CSD. As a promising technology, mNGS can rapidly identify multiple pathogens from a given source with a high degree of sensitively. Compared with conventional methods such as serological detection, PCR and bacterial culture, mNGS is a hypothesis-independent approach, which is less affected by the antibacterial treatment or the bacterial growth conditions. mNGS is particularly suited to detect the causal pathogens of rare, atypical, or complex infections. However, mNGS dose have its limitations: First, it is relatively expensive and is not effective at determining drug resistance; Second, as the human host background needs to be sequenced simultaneously with the pathogen DNA, the relative abundance of genetic material may affect the method’s sensitivity; Third, microbial contamination can easily be introduced at each test step of mNGS; Fourth, universal reference standards for clinical metagenomic assays are still lacking; and Finally, interpretation of mNGS results is an incredibly challenging. In conclusion, the clinical utility of mNGS in large-scale prospective clinical trials has not been established and further clinical validation is required.

## Data availability statement

Genome sequences were uploaded into European Molecular Biology Laboratory with accession number PRJEB52281.

## Ethics statement

This study was approved by the Ethics Committee for Scientific Research and Clinical Trials of the First Affiliated Hospital of Zhengzhou University (ID: 2022-KY-0738-002). The patient’s legal guardian provided written informed consent to participate in this study.

## Author contributions

HW conceived and designed this paper. ML reviewed and edited the manuscript. KY, PJ and EW revised it. All authors have read and approved the final manuscript.

## Conflict of interest

The authors declare that the research was conducted in the absence of any commercial or financial relationships that could be construed as a potential conflict of interest.

## Publisher’s note

All claims expressed in this article are solely those of the authors and do not necessarily represent those of their affiliated organizations, or those of the publisher, the editors and the reviewers. Any product that may be evaluated in this article, or claim that may be made by its manufacturer, is not guaranteed or endorsed by the publisher.
